# Supplementary material for: Polarity-JaM: an image analysis toolbox for cell polarity, junction and morphology quantification
Source: Nat Commun. 2025 Feb 8;16:1474. doi: 10.1038/s41467-025-56643-x (PMC11807127; doi:10.1038/s41467-025-56643-x)
Supplement: Supplementary file 1 — Supplementary Information [file 41467_2025_56643_MOESM1_ESM.pdf]

Supplementary Figures

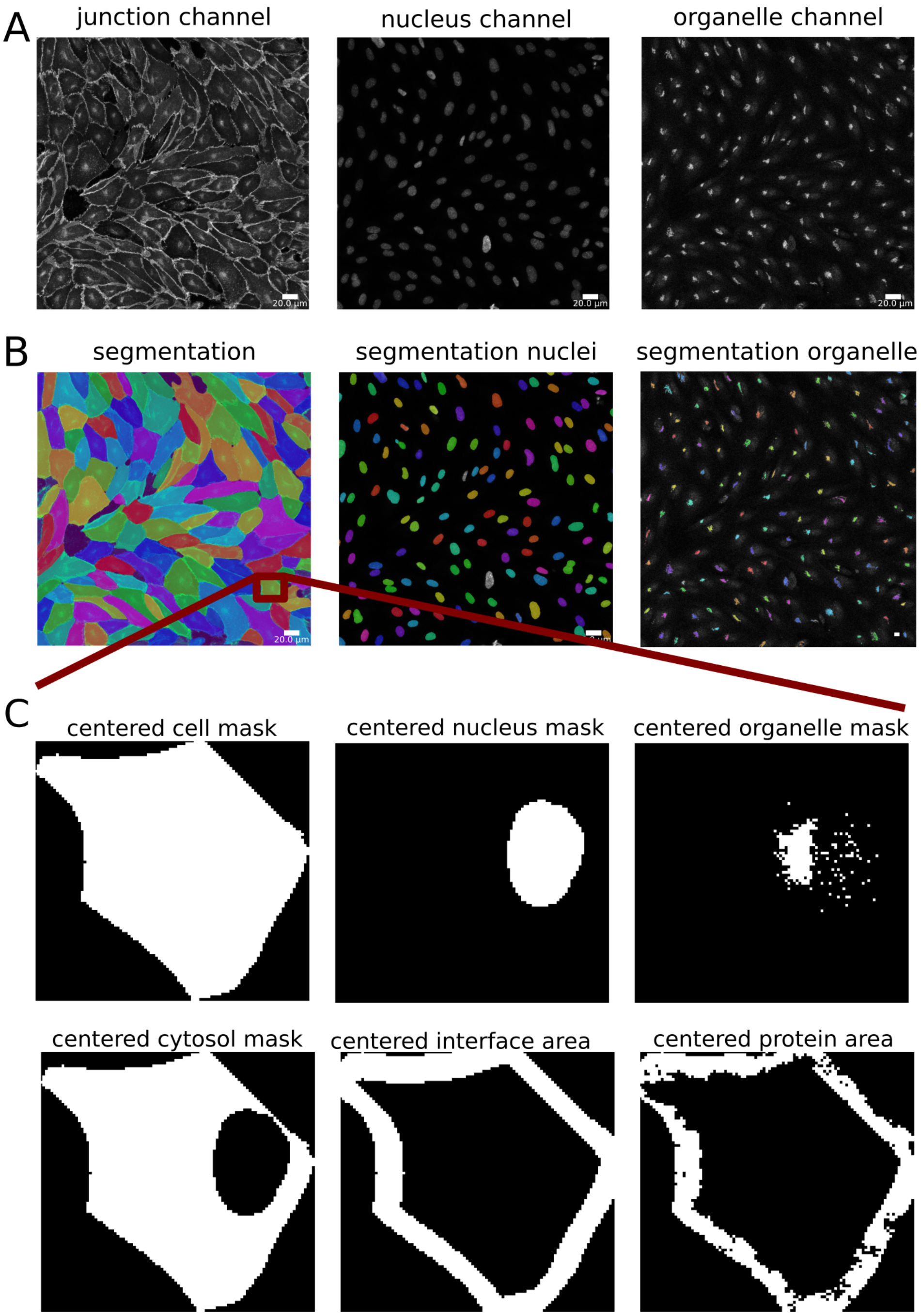

Supplementary Figure 1. Segmentation visualizations.

A: Channel information **B:** Cellpose cell and nuclei segmentation, otsu threshold on organelle channel (here: Golgi) **C:** View on a single cell, together with the nucleus, organelle (here: Golgi), cytosol, interface area, and protein area.

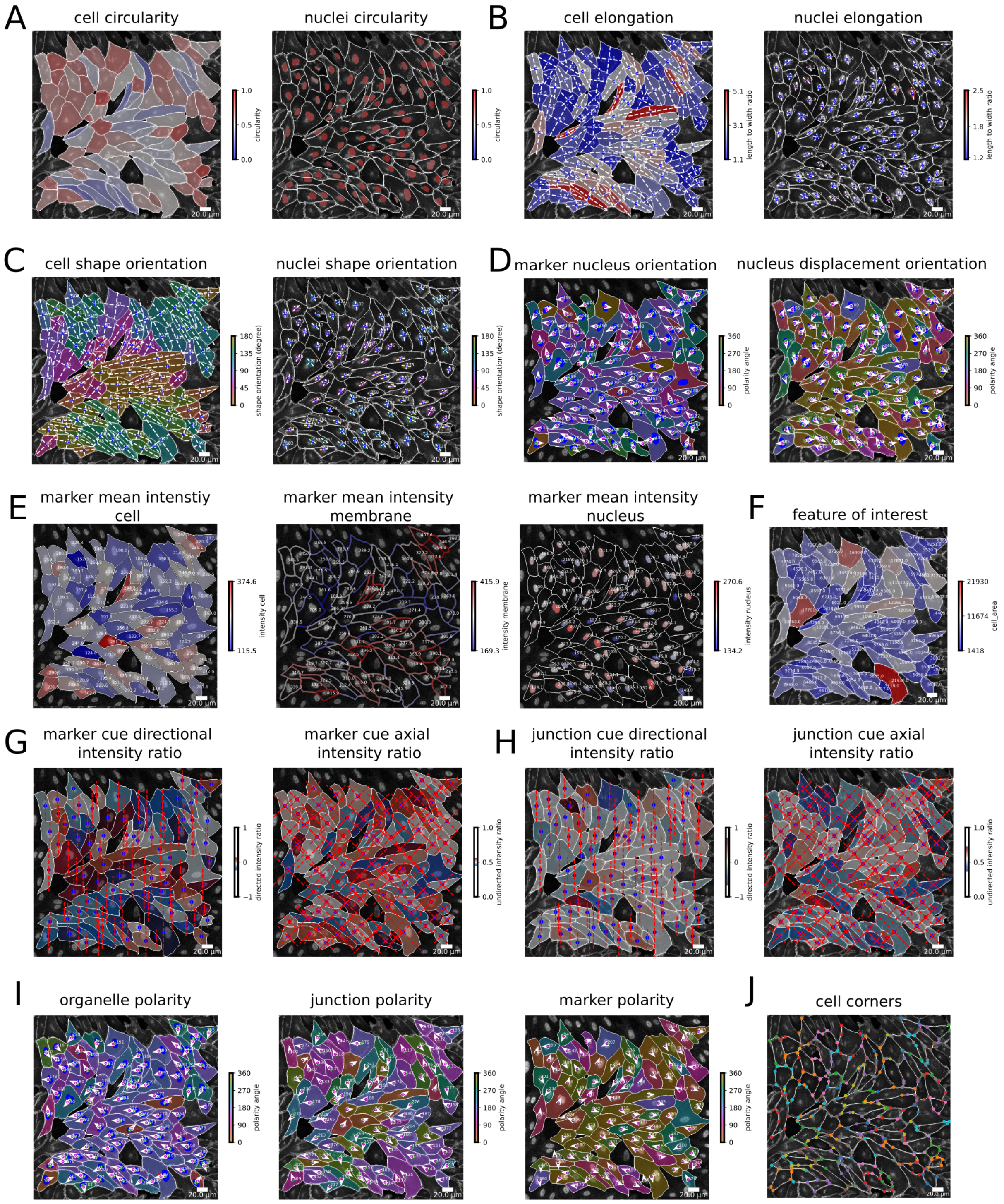

Supplementary Figure 2. Feature visualizations.

A: Circularity, **B:** Elongation, **C:** Shape orientation, **D:** Marker nucleus and nucleus displacement, **E:** Intensity information, **F:** Feature of interest (here: area) information, **G:** Marker ratio method, **H:** Junction ratio method, **I:** Polarity information for organelle (here: Golgi), junction and marker channel, **J:** Cell corners based on the Douglas-Peucker-Algorithm.

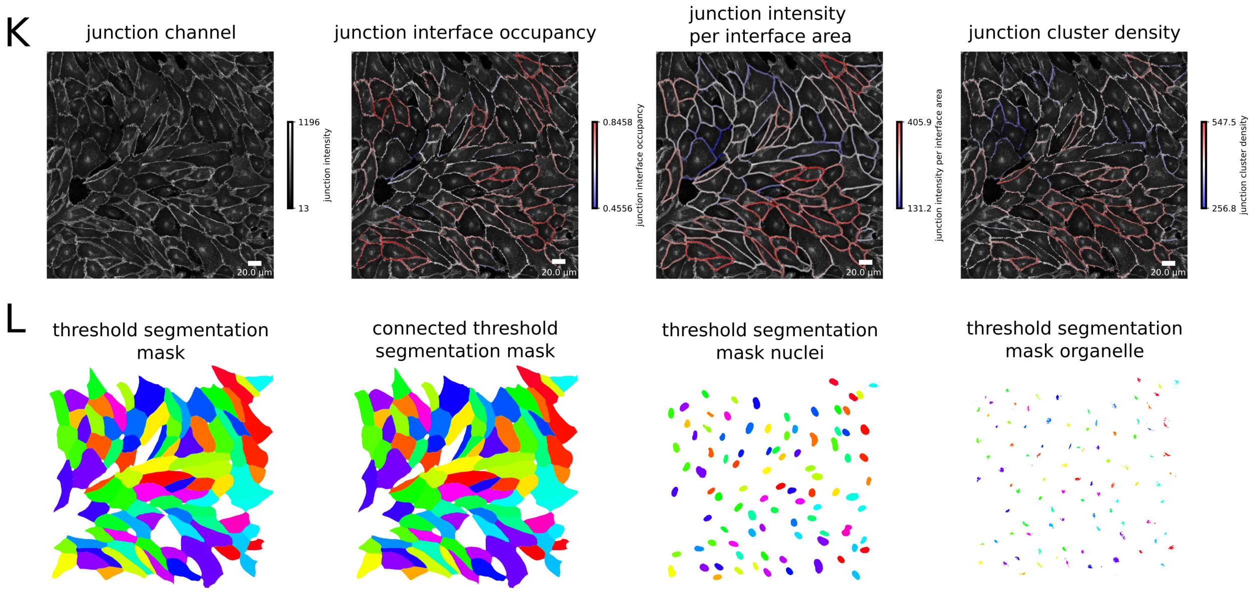

Supplementary Figure 2 cont. Feature visualisations.
K: Junction features, including channel, interface occupancy, intensity per interface area, and cluster density, **L:** Segmentation masks after applying a threshold for cell, nuclei, and organelle size.

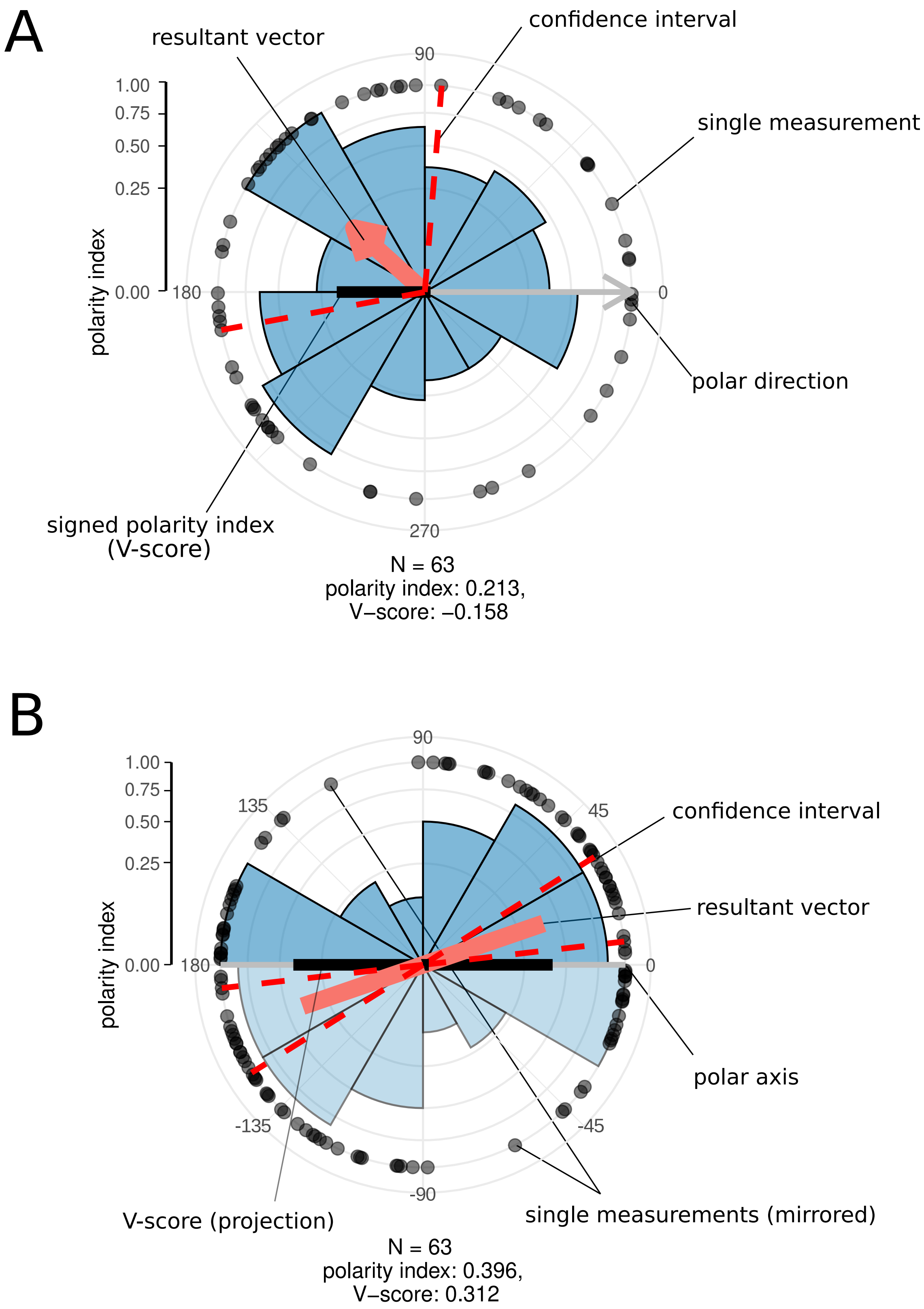

Supplementary Figure 3. Circular statistics data graphs.
Circular statistics data graphics with suggested statistical measures for **A** directional data and **B** axial data.

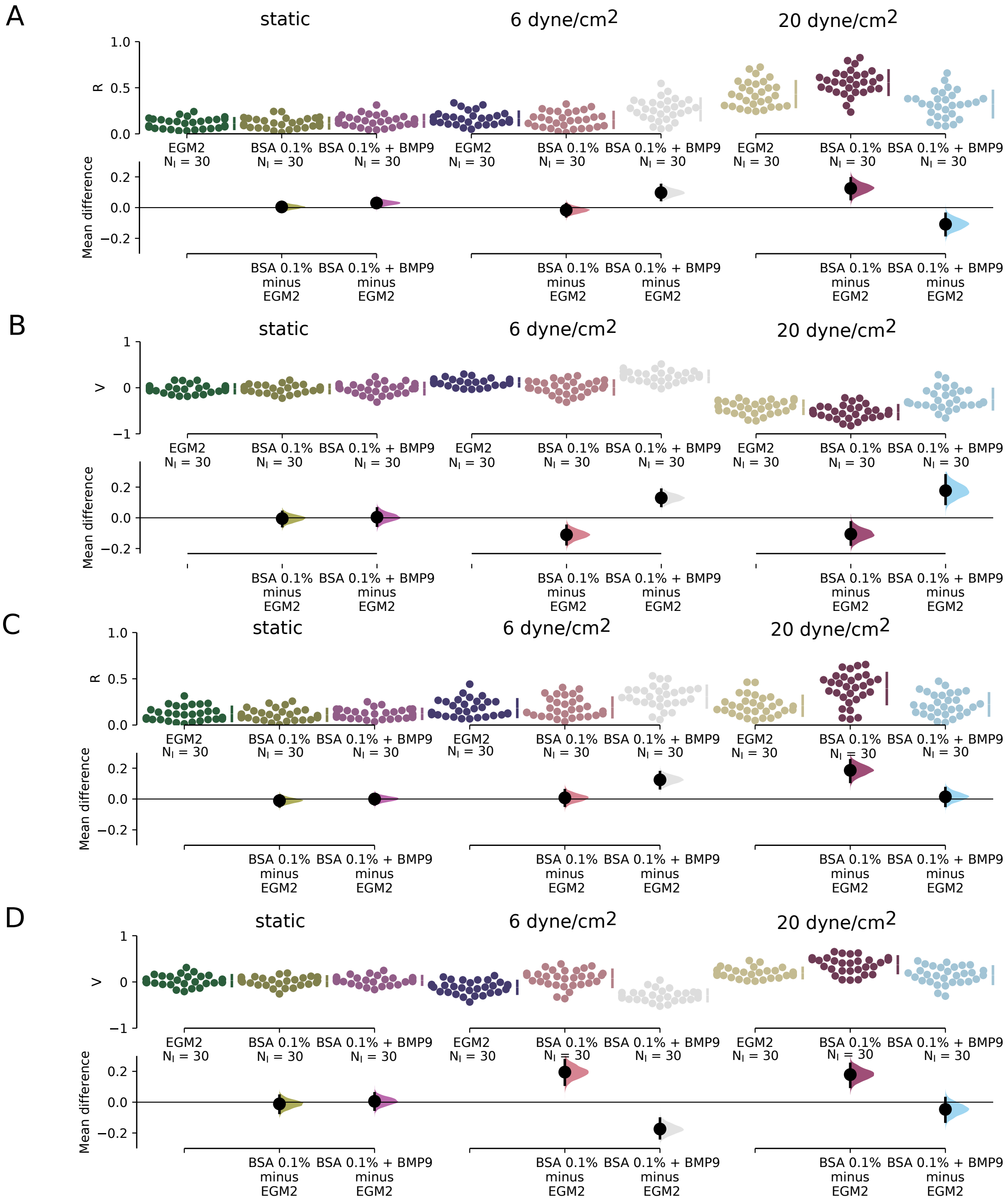

Supplementary Figure 4. Variability of polarity indices across conditions.

The data comprise $n = 3$ biological replicates per condition, with $N_I = 10$ images acquired from random positions across the entire flow slide for each replicate. As cells within individual images exhibit high spatial autocorrelation, our analysis focuses on quantifying variability across the slide, rather than within individual images. Comparison of nuclei-Golgi polarity in the indicated flow and media conditions using the **A:** polarity index (R) and the **B:** signed polarity index (V). Similarly, the comparison of the nuclei displacement polarity is made using the **C:** polarity index (R) and the **D:** signed polarity index (V). Note, that the underlying image data for this analysis is the same as in Fig. 2. Data visualization and comparison were performed using the DABEST method, with black bars representing 95% confidence intervals of the mean difference. Source data are provided as a Source Data file.

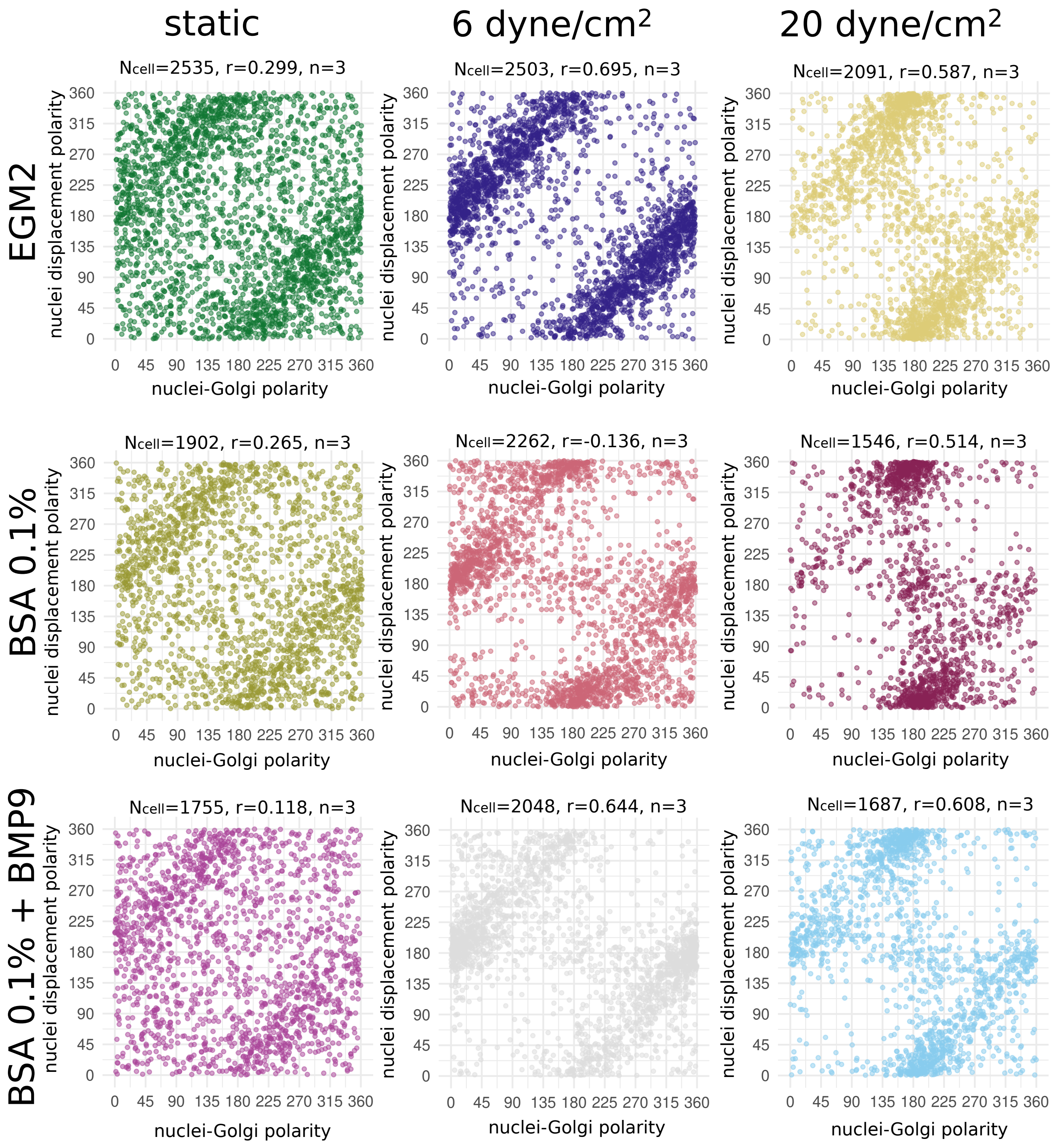

Supplementary Figure 5. Correlation of Nuclei-Golgi and Displacement Polarity.
Circular correlations between nuclei-Golgi polarity and nuclei displacement polarity for various flow and media conditions. The underlying data set is the same as in Fig. 2. Each data point represents a single cell measurement. The correlation coefficient, r, is an analogue to Pearson's correlation coefficient, adapted for circular data. Source data are provided as a Source Data file.

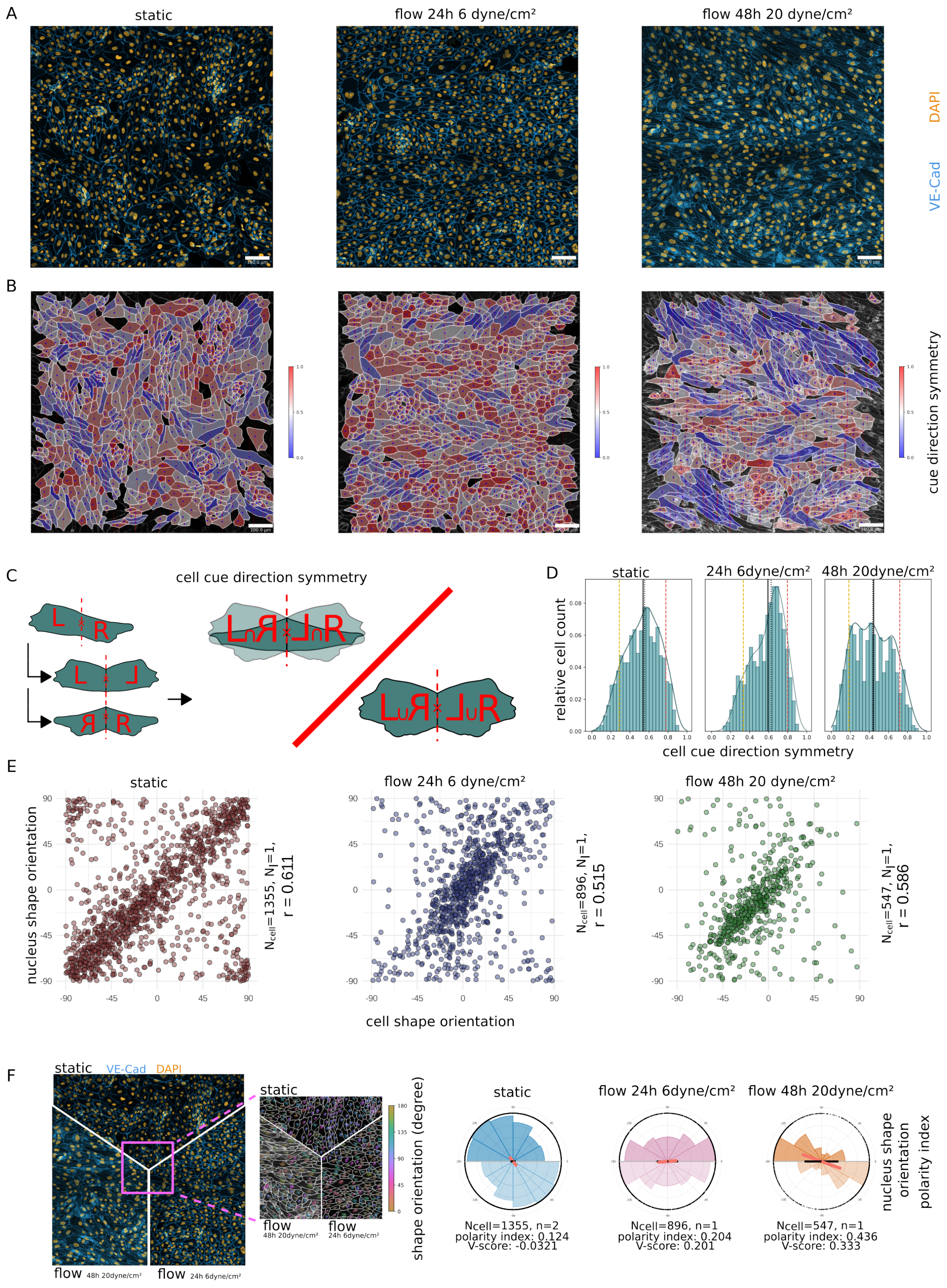

Supplementary Figure 6. Cell symmetry and nuclei/cell shape orientation.

A: Example images of endothetlial cells in static condition, exposed to 6 dyne/cm^2 shear stress over 24h, and exposed to 20 dyne/cm^2 shear stress over 48h. VE-cadherin staining is shown in blue, DAPI in yellow. **B:** Cue direction symmetry feature readout. Feature ranges from 0 to 1, where 0 indicates a high symmetry and 1 perfect symmetry. **C:** Feature sketch for cell cue direction symmetry. We divide the cell along an axis perpendicular to the cue direction through its center, mirror each half, and calculate the cue direction symmetry as the intersection over union of the resulting areas. **D:** Cue direction symmetry under static and shear stress conditions of 6 dyne/cm^2 (24h) and 20 dyne/cm^2 (48h). **E:** Correlation of nucleus shape orientation and cell shape orientation under static and shear stress conditions of 6 dyne/cm^2 (24h) and 20 dyne/cm^2 (48h). **F:** Nucleus shape orientation under static and shear stress conditions of 6 dyne/cm^2 (24h) and 20 dyne/cm^2 (48h). Source data are provided as a Source Data file.

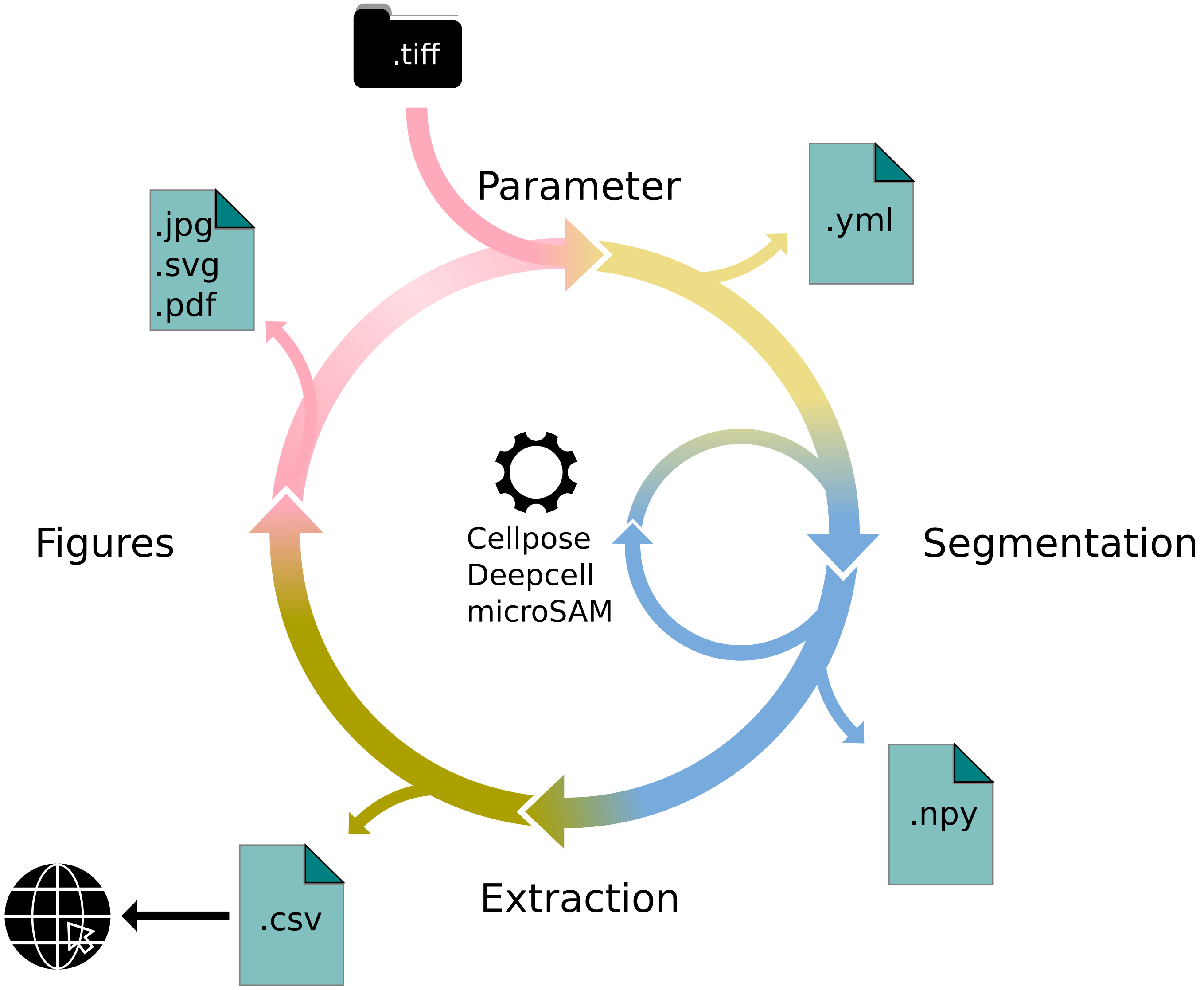

Supplementary Figure 7. Python API visual representation.
After loading the image, the user would first define parameters describing the details of the analysis. Next step would be the instance segmentation. Currently three algorithms, Cellpose, DeepCell, and microSam are supported. After the feature extraction process the visualization functionality of Polarity-JaM could be used to asses data quality.

1 Data input and structure

Often, analysts are challenged not only with the problem of actually performing the analysis, but also how and where to store the data. Iterative acquisition of images as well as various experimental settings sometimes require complex folder structures and naming scheme to organise data. Frequently, researchers face the problem of data being distributed over several physical devices, leaving them with the problem of how to execute a certain tool on a dedicated subset of images. Often a large amount of time is spent organizing data before the analysis can finally be conducted. Moreover, performing analysis steps on several experimental conditions often requires repeating the entire analysis pipeline several times to get the desired output. Another problem researchers often face is keeping track of the configuration that was used for a particular execution of software at a certain time. Sometimes this even requires some form of manual work by writing down the configuration. Polarity-JaM avoids these unnecessary chores and manual processes for the user and minimizes the time spent for reorganizing data by providing a) three run cases to cover most usage scenarios, b) a parameters yml file containing all options used for the analysis, and c) a log file fully capturing the output of the analysis.

Parameters

The parameters file is the most important argument that needs to be passed to an execution call as it specifies what analysis steps are performed and hence what output will be created. The file additionally is copied to the output folder and hence allows the user to fully reproduce and comprehend the analysis at a later time-point. An overview of all possible keywords is given in Table 10. This includes configuration parameters describing the image (image parameter), analysis (runtime parameter), and visualisation (plot parameter). Please note that the parameters necessary for the segmentation algorithm are listed separately in Table 14.

Run Options

To tackle the problem of various examples of use, Polarity-JaM offers three run options. The mode *run* can be used for a single image and only requires an input image, and an output path. *run-stack* should be chosen if a set of images in a folder needs to be processed. Instead of a single image, a folder can be specified. Lastly, the *run-key* option needs a csv file describing the storage structures and experimental conditions and must be passed as an argument. The structure of the csv is deliberately kept simple and is shown in table 9. Paths are relative to an input path that can be passed separately as an argument to the execution call. Hence, the data can be copied to a different physical device and the analysis can be repeated without altering the csv file as long as the underlying data structure remains untouched.

2 Features

The feature extraction pipeline is the process of extracting all relevant features from an input image. This is a complex process that can be separated in three major parts. First, the image is segmented, segregating each cell such that in the second step its features can be extracted. In the third and last step, a graph structure is build and a neighborhood analysis performed. To the time writing the manuscript, the user can choose between three segmentation algorithms: Cellpose(9), microSAM(8) a fine-tuned model based on sam(7), and DeepCell(6).

The pipeline produces various outputs, depending on the parameter configuration and input provided. In general, generated output can be differentiated in the two categories a) features, and b) visualization. Features are gathered in a csv file containing the individual cells as rows and their corresponding feature values as columns. Visualizations for the extracted features can be optionally created and written to disk. These plots should be used for quality control before continuing downstream analysis of the extracted features in the web application. An example of all available visualizations is shown in Supplementary Figure 2.

Feature Categories

Our Toolbox focuses on features we categorized in 5 different categories: Identification and localization, morphology, polarity and intensity. Our manuscript mainly focuses on the first 4, but for completion we report the fifth category as well.

Localization and identification features are used to identify and locate the object of interest in the image. These features are essential for the subsequent analysis of the image data and are a direct result of the image segmentation. Morphological features characterize the shape and size of the object of interest. These can be further classified

into geometric features, which describe the object's overall shape; structural features, which detail the internal structure; and ratio-based features, which involve calculations of various proportions. Polarity features characterize the orientation and symmetry of the object of interest. Broadly, these can be divided into directional features, which describe the orientation of two objects (or rather two points) relative to one another, and axial features, which define the orientation of an object relative to a reference axis. Therefore, directional and axial polarity features depend on localization and morphological outcomes. Additionally, predefined axial asymmetry features quantify intensity asymmetry relative to a designated reference axis (or several) by calculating various proportions between the corresponding areas. These features base primarily on intensity values present in a certain channel and region, but involve the definition of an axis and thus are based on polarity in nature. We defined intensity features as its own category. They characterize the object's intensity and expression levels within different regions and they can be summed, averaged, or compared using ratios or proportions. Lastly, we report features belonging to the topology of the image. They describe the spatial relationships between objects in an image. These features are useful for analyzing the distribution of objects and their interactions within the image. Specifically, we focus on the connectivity (neighborhood) and spatial arrangement properties of the objects in form of a Moran's I statistical analysis. For a full overview of available categories we refer to table 1.

Targets

Features can be extracted for multiple targets, which will be explained shortly. Depending on the configuration, specific targets may be included or excluded from the extraction process. Supplementary Table 2 lists the targets along with their required configurations. Features that target a single cell mainly comprise morphological features such as area, perimeter and eccentricity and are shown in Supplementary Table 6. Optionally,the *nucleus*, *organelle*, *marker*, and the *junction* can be targeted and their specific features are explained in tables 4, 5, 7 and 8 respectively. Nucleus features mainly comprise morphological features, such as their size, position, orientation. Organelle features contain positional, but also distance information to the nucleus, whereas marker features include besides positional, also mean expression values of several cell regions. (e.g. membrane, nucleus and cytosol). Junction features mainly comprise ratio values between different areas of the junction region of the cell membrane (11), but also mean intensity information and polarity. Additionally, Polarity-JaM offers the possibility to target the neighborhood of a cell and are gathered as *neighborhood features*. An overview is found in Supplementary Table 12. Given a feature of interest (e.g. cell size) the Morans I correlation analysis can be performed. *group_features* are depicted in Supplementary Table 13. Please note that their values address an entire image and not a single cell and should be interpreted accordingly.

3 Visualizations

Beside features, several visualizations are created during the analysis. They mainly serve as quality control. To the point of manuscript writing Polarity-JaM provides 19 different visualizations:

- image channel intensity information
- segmentation masks
- threshold segmentation masks
- single cell closeup
- cell corners
- feature of interest
- cell and nuclei elongation
- cell and nuclei circularity
- cell and nuclei shape orientation
- cell cue direction symmetry
- nucleus displacement orientation
- organelle polarity
- nucleus marker orientation
- marker polarity
- marker expression
- marker cue intensity ratio
- junction features
- junction polarity
- junction cue intensity ratio

Quality control of the segmentation output is a crucial step for downstream analysis. For this purpose, the feature extraction pipeline creates for every input image a plot showing the given channel configuration used for segmentation together with the segmentation outcome (Supplementary Figure 1 A,B). Optionally, a closeup view for each cell can be plotted as shown in Supplementary Figure 1 C. Feature visualizations are shown in Supplementary Figure 2 and highly depend on the quality of the segmentation. We highly recommend to perform a quality control of the segmentation result before continuing downstream analysis for example with the Polarity-JaM web app.

4 API Usage

Polarity-JaM offers the possibility to completely run an anaylsis within a Jupyter Notebook. Several example notebooks can be found in the GitHub repository https://github.com/polarityjam/polarityjam/tree/main/docs/notebooks focusing on various aspects of an analysis. This includes the basic usage of Polarity-JaM to perform a feature extraction to using Polarity-JaM as a python library for enhanced image analysis. We now shortly describe a basic analysis in chronological order. First, the user loads the image. Second, the user sets up the parameters for i) the image(s), ii) the runtime (analysis procedure), including the choice of the segmentation algorithm, and optionally iii) the visualizations. Additionally, parameters will be saved on disk in yml format to support replicability. Third, the previously set segmentation algorithm is loaded, initially with its default parameters. At this point the user has the option to alter these before performing the segmentation. Any segmentation algorithm that is currently supported in Polarity-JaM requires two steps for the user to perform: i) preparing the image for segmentation, and ii) using the prepared image to perform the segmentation. The division in two steps is specifically designed to facilitate visual quality inspection by using the visualization functionality of Polarity-JaM. Additional segmentation algorithms (other than Cellpose) are implemented with the help of Album (67), a decentralized distribution platform where solutions (in this case implementations of segmentation algorithms) are distributed with their execution environment. This allows to easily switch to a different segmentation algorithm when performance is poor. The overhead of installing the algorithm in its correct environment is taken from the user. Regardless of the used algorithm the analysis steps follow the same semantic (e.g. the same function call) such that usage of an unknown algorithm is facilitated. Output of a segmentation procedure is always an instance segmentation mask in numpy format. We provide detailed information in a Jupyter Notebook that can be found under the following link: https://github.com/polarityjam/polarityjam/blob/main/docs/notebooks/polarityjam-notebook_seg.ipynb To improve downstream analysis, instance masks for nuclei and organelle should additionally be calculated whenever the corresponding information is present in the image. To facilitate the process for the user, every segmentation algorithm supported in Polarity-JaM has a mode that can be specified in the segmentation step. Currently, there are three modes available: "nucleus", "cell", and "organelle". Please note that not every algorithm supports all modes. The user is however free to entirely skip any segmentation step and provide instance segmentation masks elsewhere produced. As a fourth step, the user performs the actual feature extraction by first setting up an initially empty collection that can be then passed to the routine performing the extraction, together with the instance mask(s), the original image, and the parameter describing the image. Gathering features in a collection allows the user to potentially collect features of various images for example by looping over images in a given folder. Last step should always be to use the visualization functionality of Polarity-JaM to assess quality and get a first impression of selected features before moving on to the downstream analysis of the features in the Polarity-JaM Web-App. The entire API is depicted in Supplementary Figure 7 and shows the workflow the user performs when working in a Jupyter Notebook.

5 Circular statistics

A broad range of scientific studies involve taking measurements on a circular, rather than a linear scale (often variables related to orientations or circadian times). However, their analysis is not straightforward and requires special statistical tools. All features in our study were classified into periodic and linear features. Among periodic features, we further distinguish directional features with values in a full circular scale, meaning that the data repeat after 360 degrees (or 2 π in radians) and axial features that repeat after 180 degrees (or π in radians). Axial data refer to an axis, in our case the long axis of the cell or nucleus shape, rather than to a direction.

Circular data presents some unique challenges for statistical analysis because traditional statistical methods may not be appropriate for this type of data. For example, computing the average or mean of circular data by summing the values and dividing by the number of observations can produce incorrect results. Therefore, specialised statistical methods have been developed to analyse circular data, such as circular statistics and directional statistics. These methods take into account the periodic nature of the data and can provide a more accurate result. All the different features such as nuclei-Golgi polarity, cell shape orientation, cell elongation, or junction properties can be correlated amongst each other compared and correlated to linear read-outs such as cell size or cell elongation on a single cell level.

Several of the extracted polarity features are periodic and require different means of statistical comparison. The

polarity index is defined as follows

$$\mathbf{r}_i = \begin{pmatrix} \cos\alpha_i \\ \sin\alpha_i \end{pmatrix} \tag{S1}$$

and the average resultant vector

$$\mathbf{r} = \frac{1}{N}\sum_{i=1}^{N}\mathbf{r}_i \tag{S2}$$

.

Although the polarity index describes the concentration of the distribution with respect to the mean direction, we may also be interested in a measure that quantifies the degree of orientation towards a given direction α_p, which is referred to as the polar direction (28). This is described by the V-score, which is computed from

$$V = cR, \tag{S3}$$

$$\text{with } c = \cos(\bar{\alpha} - \alpha_p). \tag{S4}$$

Therefore, V is equal to the polarity index, $V = R$ if aligned with the given direction and takes the negative value if aligned with the given direction $V = -R$.

These statistical measures can also be applied to axial data, converting these data to directional data by doubling all values $\theta_i = 2\phi_i$. The mean direction was calculated from $\bar{\phi} = \frac{\theta}{2}$, where $\bar{\theta}$ is the common circular mean of the directional data θ_i. Similarly, the polarity index (PI) was calculated as the length of the mean resulting vector of directional values θ_i. Again, a PI value varies between 0 and 1 and indicates how much the distribution is concentrated around the mean. A value of PI close to 1 implies that the data are concentrated around the mean direction, while a value close to 0 suggests that the data are evenly distributed or random. The axial V-score is computed from

$$V = cR, \tag{S5}$$

$$\text{with } c = \cos(\bar{\theta} - 2\phi_p), \tag{S6}$$

where θ_p is a given polar orientation that is a priori known. Note, that in this case, the V-score is not the projection of the mean resultant $\mathbf{r}$. However, we can compute the following relationship. We assume

$$\tilde{\phi}_p = \phi_p \bmod \pi \tag{S7}$$

and compute

$$c_p = \cos(\bar{\alpha} - \tilde{\phi}_p). \tag{S8}$$

With the commonly known trigonometric identity $\cos(2\alpha) = 2\cos^2(\alpha) - 1$, we obtain:

$$c = \cos(\bar{\theta} - 2\phi_p) \tag{S9}$$

$$= \cos(2\bar{\alpha} - 2\phi_p) \tag{S10}$$

$$= \cos^2(\bar{\alpha} - \phi_p) - 1 \tag{S11}$$

$$= \cos^2(\bar{\alpha} - \tilde{\phi}_p) - 1 \tag{S12}$$

$$= c_p^2 - 1. \tag{S13}$$

The projection c_p takes values between 0 and 1, with 1 in the case of perfect alignment with the given polar direction and zero for random or perpendicular alignment with the polar direction.

Our web application provides the most common statistical tests for different scenarios, including the Rayleigh test, the V-test, the Rao spacing test and the Watson test applied to directional and axial data (transformed as above). For further discussion of statistical analysis of circular data and extension to comparative statistical analysis of circular data, we refer to (28, 29) and also consider more recent studies (31).

We also provide a computation for measures of association. The correlation coefficient ρ_{cc}, which quantifies the relationship between two directional variables $\alpha = (\alpha_1, \dots, \alpha_N)$ and $\beta = (\beta_1, \dots, \beta_N)$, is calculated as follows (29):

$$\rho_{cc} = \frac{\sum_i \sin(\alpha_i - \bar{\alpha})\sin(\beta_i - \bar{\beta})}{\sqrt{\sum_i \sin^2(\alpha_i - \bar{\alpha})\sin^2(\beta_i - \bar{\beta})}}. \tag{S14}$$

The relationship between a directional random variable α and a linear variable x is evaluated by individually correlating x with $\cos(\alpha)$ and $\sin(\alpha)$. For this purpose, we establish the correlation coefficients $r_{sx} = c(\sin(\alpha), x)$, $r_{cx} = c(\cos\alpha, x)$, and $r_{cs} = c(\sin(\alpha), \cos(\alpha))$, where $c(x,y)$ denotes the Pearson correlation coefficient. Following this, the circular-linear correlation ρ_{cl} is defined as

$$\rho_{cl} = \sqrt{\frac{r_{cx}^2 + r_{sx}^2 - 2r_{cx}r_{sx}r_{cs}}{1 - r_{cs}^2}}. \tag{S15}$$

For axial-axial, axial-circular and axial-linear correlations, we transform the axial data as above by doubling all values to obtain the correlation coefficient.

Supplementary Tables

Localization Features				
Input	Feature or Filename suffix	Sub-Category	Category Sketch	Interpretation
image	__seg.npy<br>__seg__nuc.npy	label	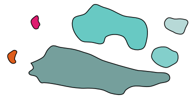	mask of objects of the image.
label	__x<br>__y	centroid	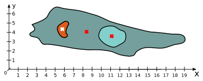	x and y coordinates of the centroid of the given segmentation mask
label + channel	__centroid__x<br>__centroid__y	weighted centroid	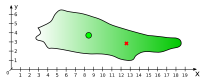	x and y coordinates of the weighted centroid of a given segmentation mask, together with an intensity channel
Morphology Features				
Input	Feature suffix	sub-category	Category Sketch	Interpretation
label	__area<br>__axis__length<br>__perimeter<br>__distance	geometric	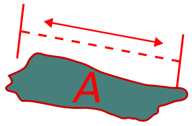	describes the object's overall shape
label	__eccentricity	structural	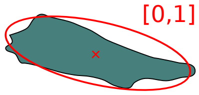	the internal structure of the object
label	__ratio	ratio	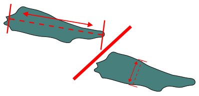	Shape components relative to each other.
Polarity Features				
Input	Feature suffix	sub-category	Category Sketch	Interpretation
label	__orientation__rad<br>__orientation__deg	directional	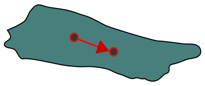	Vector of one point to another is computed, the angle and length of orientation of the vector are extracted.
label	__orientation__rad<br>__orientation__deg	axial	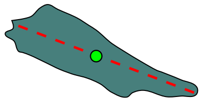	Orientation of the object shape computed from image moments.
label	__axial__intensity__ratio<br>__directional__intensity__ratio	predefined axis asymmetry	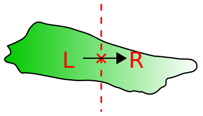	Left to right asymmetry with respect to a pre-defined direction (black arrow). The cell is split along the cell centroid.

Supplementary Table 1. Overview of feature categories.

Intensity Features				
Input	Feature suffix	sub-category	Category Sketch	Interpretation
label + intensity channel	_mean_expression	mean	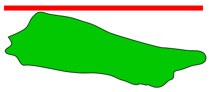	Mean Intensity of an object over a given intensity channel.
label + intensity channel	_sum_expression	sum	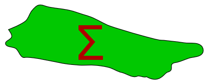	Absolute summed intensity value of an object over a given intensity channel
label + intensity channel	_intensity_ratio	ratio	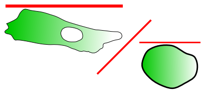	Mean intensity of one object relative to another object usually contained within it.
Topology Features				
Input	Feature prefix	sub-category	Category Sketch	Interpretation
label	neighbors_	connectivity	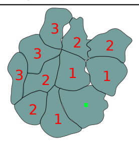	Connectivity of one cell.
label + intensity channel	moran_	spatial arrangement	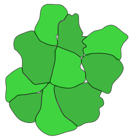	Spacial arrangement of a certain feature over all identified cells.

Supplementary Table 1 cont. Continued: Overview of feature categories.

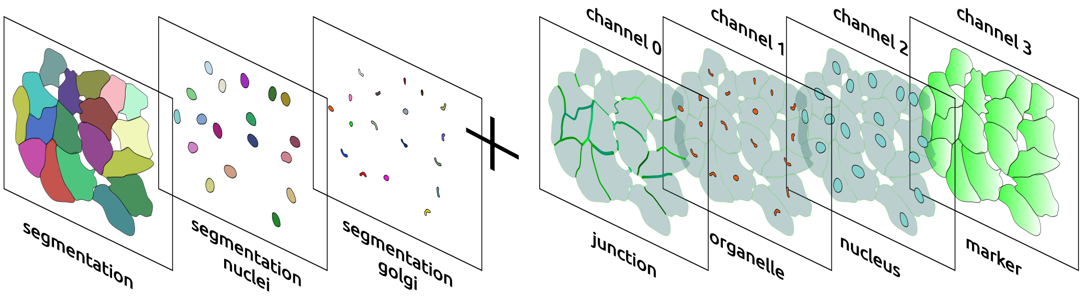

Target name	Target sketch	Required configuration	Description
single cell	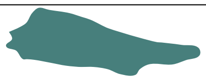	segmentation	The general features extracted from the image.
nucleus	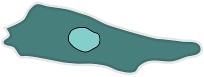	cell + nucleus segmentation + nucleus channel	All features belonging to the nucleus of the cell.
organelle	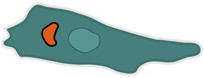	cell + organelle segmentation + organelle + nucleus channel	All features belonging to the organelle of a cell. Note that several features additionally require a nuclei staining.
junction	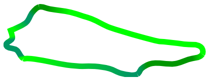	cell segmentation + junction channel	All features belonging to the junctions of a cell.
marker	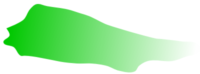	cell segmentation + expression marker channel	All features belonging to the expression marker.
marker nucleus	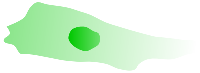	cell + nucleus segmentation + expression marker + nucleus channel	All features belonging to the expression marker and nucleus.
marker cytosol	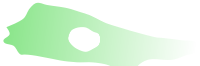	cell + nucleus segmentation + expression marker + nucleus channel	All features belonging to the expression marker and cell without nucleus.
cell neighborhood	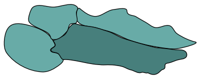	cell segmentation + FOI	Statistical properties of a feature of interest (FOI). Includes neighborhood statistics. Default FOI is "area".
group	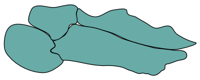	cell segmentation + FOI	Group properties. Image wise. Includes Morans I correlation analysis of the feature of interest (FOI). Default FOI is "area".

Supplementary Table 2. Overview of the requirements (configuration) met to extract features for a given target. Note that when a segmentation is not provided it will be calculated.

	Polarity-JaM	Quantify-Polarity	Junction-Mapper	Chesnais et al. 2022
segmentation	flexible, Cell-pose (Deep Learning model) native	adapted water-shed algorithm	Filter-based	Cell Profiler workflow
organelle asymmetries	nuclei-Golgi orientation, polarity index	no	no	no
shape asym-metry	size, shape, eccentricity, orientation, topology	size, shape, eccentricity, orientation, topology	no	area, perime-ter, shape descriptors and cell neigh-bours
signaling gradients & marker expression	mean intensity in the cytosol, nuclei, and on the cell mem-brane as well as their ratios	no	no	Notch inten-sity
junction morphology	intensity of junctional markers	intensity of junctional markers	morphology and intensi-tyfeatures	unsupervised classification of junctional morphology of arterial, venous, and microvascular endothelial cell populations
polarity measures	2-axial polar-ity based on in-tensity of junc-tional markers	nuclei-Golgi; polarity of cytosolic and junctional markers, 2-axial cell and nuclei shape orientation	no	no

Supplementary Table 3. Comparison of available tools QuantifyPolarity (20), JunctionMapper (11) and Chesnais et al. (16) and our tool.

Feature Name	Category	Feature	Description
nuc_x	localization	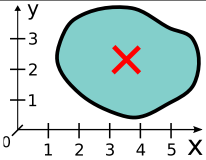	X position (horizontal axis) of the cell nucleus.
nuc_y	localization	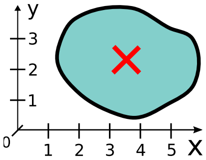	Y position (vertical axis) of the cell nucleus.
nuc_disp_orientation_rad	polarity	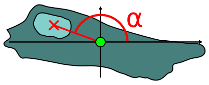	The displacement orientation of the nucleus from the center of the cell in rad
nuc_disp_orientation_deg	polarity	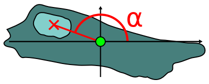	The displacement orientation of the nucleus from the center of the cell in deg
nuc_shape_orientation	polarity	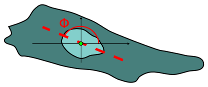	Long axis of ellipsoid fit of the nucleus.
nuc_major_axis_length	morphology	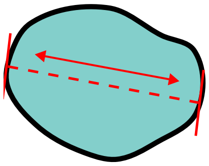	The length of the major axis of the nucleus.
nuc_minor_axis_length	morphology	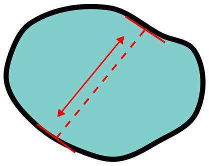	The length of the minor axis of the nucleus.
nuc_area	morphology	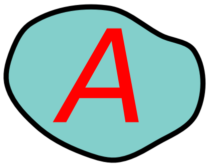	The area of the nucleus.
nuc_perimeter	morphology	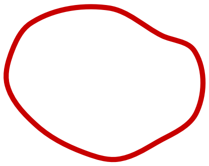	The perimeter of the nucleus.
nuc_eccentricity	morphology	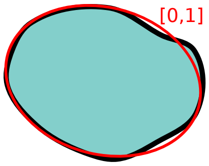	Value for the elongation of the nucleus. Between 0 and 1, where 0 corresponds to a perfect circular nucleus and 1 to a strongly elongated nucleus.
nuc_major_to_minor_ratio	morphology	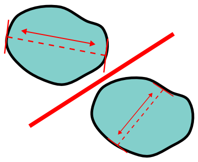	Ratio between the major and the minor axis of the nucleus.

Supplementary Table 4. Features targeting the nucleus. Note that a nucleus channel must be configured in the parameters file.

Feature Name	Category	Feature	Description
organelle_x	localization	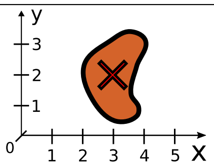	The X coordinate (horizontal axis) of the center of the cell organelle.
organelle_y	localization	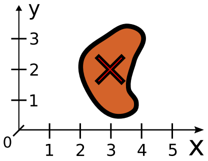	The Y coordinate (vertical axis) of the center of the cell organelle.
organelle_distance	morphology	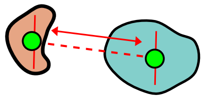	Distance from cell organelle to the nucleus.
organelle_orientation_rad	polarity	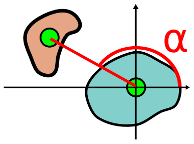	The orientation in rad of the organelle to the nucleus
organelle_orientation_deg	polarity	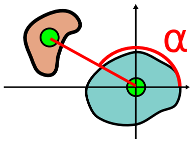	The orientation in deg of the organelle to the nucleus

Supplementary Table 5. Features targeting the organelle. Note that an organelle channel must be specified.

Feature Name	Category	Feature	Description
filename	None		The filename where the cell was found.
img_hash	None		The sha1 hexadecimal hash of the image content.
label	identification	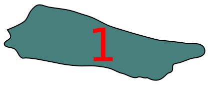	The segmentation label of the particular cell.
cell_x	localization	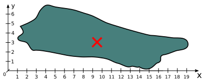	The X coordinate (horizontal axis) of the center of the cell.
cell_y	localization	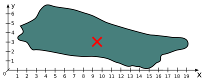	The Y coordinate (vertical axis) of the center of the cell.
cell_shape_orientation	polarity	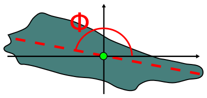	Long axis of ellipsoid fit of the cell.
cell_cue_direction_symmetry	polarity	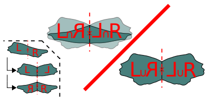	Cell shape symmetry that takes into account a predefined cue direction. Value between 0 and 1. 0 indicates a total shape asymmetry, 1 a perfect symmetric shape along the flow.
cell_major_axis_length	morphology	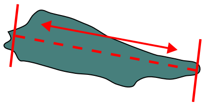	Length of the major axis of the cell.
cell_minor_axis_length	morphology	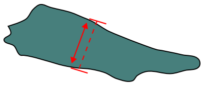	Length of the minor axis of the cell.
cell_eccentricity	morphology	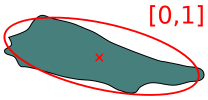	Value for the elongation of the cell. Between 0 and 1, where 0 correspond to a perfect circular cell and 1 for a strongly elongated cell.
cell_major_to_minor_ratio	morphology	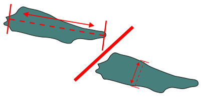	Ratio between the major and the minor axis of the cell.
cell_area	morphology	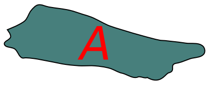	The area of the cell.
cell_perimeter	morphology	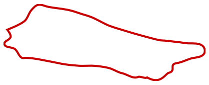	The perimeter of the cell.

Supplementary Table 6. Features targeting a single cell.

Feature Name	Category	Feature	Description
marker_mean_expr	intensity	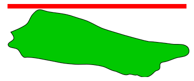	Mean expression of the channel with the marker.
marker_sum_expr	intensity	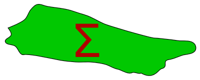	The absolute sum of the expression of the marker.
marker_centroid_x	localization\intensity	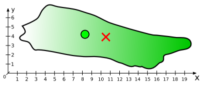	The X coordinate (horizontal axis) of the center of the marker expression.
marker_centroid_y	localization\intensity	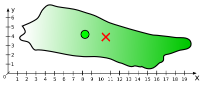	The Y coordinate (vertical axis) of the center of the marker expression.
marker_centroid_orientation_rad	polarity	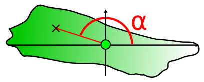	Intrinsic asymmetry of the cell, reported in radian.
marker_centroid_orientation_deg	polarity	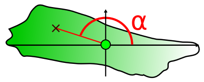	Intrinsic asymmetry of the cell, reported in degree.
marker_mean_expression_mem	intensity	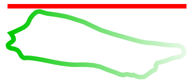	Mean membrane expression.
marker_sum_expression_mem	intensity	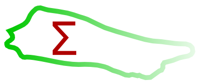	The absolute sum of the membrane expression.
marker_mean_expression_nuc	intensity	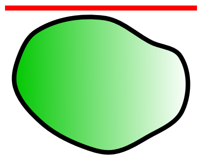	The mean expression of the nucleus.
marker_sum_expression_nuc	intensity	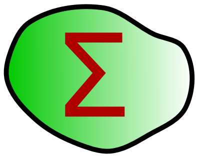	The absolute sum of the nucleus expression.
marker_mean_expression_cyt	intensity	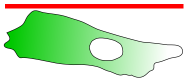	The mean expression of the cell cytosol.
marker_sum_expression_cyt	intensity	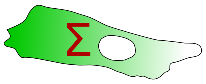	The absolute sum of the cell cytosol expression.
marker_nucleus_orientation_rad	polarity	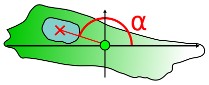	The orientation in rad of the marker centroid to the nucleus.
marker_nucleus_orientation_deg	polarity	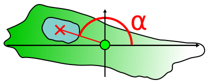	The orientation in rad of the marker centroid to the nucleus.
marker_mean_expression_nuc_cyt_ratio	intensity	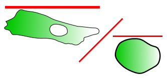	The ratio between the mean marker expression in the region of the nucleus and the mean marker expression in the cytosol.
marker_cue_directional_intensity_ratio	polarity	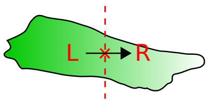	The ratio of the left vs. right cell membrane intensity in cue direction.
marker_cue_axial_intensity_ratio	polarity	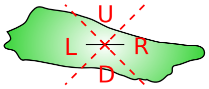	The ratio of the sum of cell membrane quarters in cue direction and the total membrane intensity.

Supplementary Table 7. Features targeting a marker channel. Note that a marker channel needs to be configured.

Feature Name	Category	Feature	Description
junction__centroid__x	localization\intensity	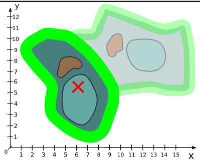	The X coordinate (horizontal axis) of the centre of the junction expression.
junction__centroid__y	localization\intensity	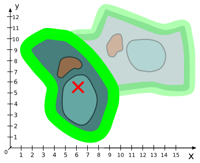	The Y coordinate (vertical axis) of the centre of the junction expression.
junction__perimeter	morphology	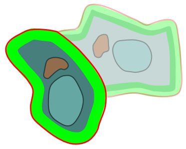	The perimeter of the junction area.
junction__protein__area	morphology	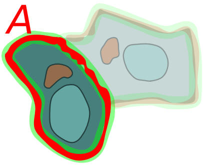	The area with junction protein expression.
junction__mean__intensity	intensity	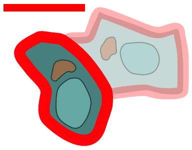	The mean junction intensity value.
junction__protein__intensity	intensity	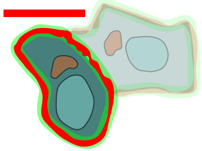	The mean protein intensity by area.
junction__interface__linearity__index	morphology	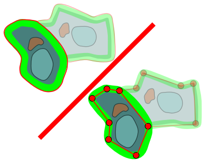	The linearity index of the junction.
junction__interface__occupancy	morphology	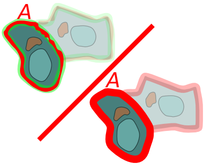	The ratio between junction area and junction protein area.
junction__intensity__per__interface__area	morphology\intensity	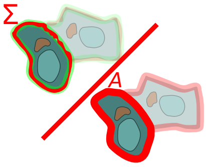	The ratio between the junction protein intensity and the junction area.
junction__cluster__density	morphology\intensity	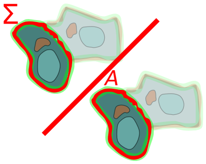	The ratio of junction protein intensity and junction protein area

Supplementary Table 8. Features that can be gathered if a junction channel is configured.

Feature Name	Category	Feature	Description
junction_centroid_orientation_rad	polarity	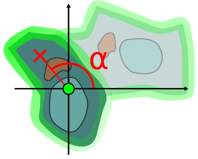	The orientation in rad of the junction intensity area centroid to the centre of the cell.
junction_centroid_orientation_deg	polarity	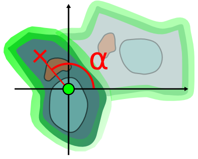	The orientation in deg of the junction intensity area centroid to the centre of the cell.
junction_cue_directional_intensity_ratio	polarity	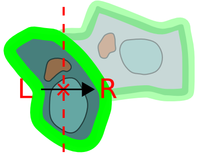	The ratio of the left vs. right cell membrane intensity in cue direction.
junction_cue_axial_intensity_ratio	polarity	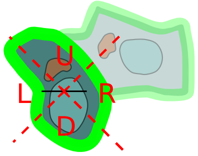	The ratio of the sum of cell membrane quarters in cue direction and the total membrane intensity.

Supplementary Table 8 cont. Cont. Features that can be gathered if a junction channel is configured.

folder_name	condition_name	replicate
folder_1	condition_1	rep_1
folder_2	condition_1	rep_2
folder_3	condition_1	rep_3
folder_4	condition_2	rep_1
folder_5	condition_2	rep_2

Supplementary Table 9. Structure of the *key_file* that can be used to specify experimental conditions and data structure. Note that given folder_names are relative to a root folder passed with the argument named *in_path*.

Category	Parameter	Options	Description
image	channel_junction	-1,0,1,2,3,...	Specifies which channel in the input image(s) holds information about the junction signals. -1 to indicate there is no channel.
	channel_nucleus	-1,0,1,2,3,...	Specifies which channel in the input image(s) holds information about the nucleus. -1 to indicate there is no channel.
	channel_organelle	-1,0,1,2,3,...	Specifies which channel in the input image(s) holds information about the organelle (e.g Golgi apparatus). -1 to indicate there is no channel.
	channel_expression_marker	-1,0,1,2,3,...	Specifies which channel in the input image(s) holds information about the expression marker. -1 to indicate there is no channel.
	pixel_to_micron_ratio	float	Specifies the resolution of the image.
runtime	min_cell_size	integer	Minimal expected cell size in pixel. Threshold value for the analysis. Cells with a smaller value will be excluded from the analysis.
	min_organelle_size	integer	The minimal diameter of the organelle. Threshold value for the analysis. Cells with an organelle with a smaller value will be excluded from the analysis.
	min_nucleus_size	integer	The minimal diameter of the nucleus size. Threshold value for the analysis. Cells with a nucleus with a smaller value will be excluded from the analysis.
	membrane_thickness	integer	Expected membrane thickness in pixel.
	junction_threshold	float	Threshold for junction intensity mask. If not set, automatically calculated via otsu thresholding.
	feature_of_interest	area	Name of the feature for which a neighborhood statistics should be calculated. Any feature can be used here. Look at [the output section](#fep_out) to see all available options.
	dp_epsilon	integer	Epsilon value for the Douglas-Peucker corner detection algorithm. Determines the maximal perpendicular distance between two points to be considered a corner.
	cue_direction	integer	The cue direction along which singaling gradients are calculated. Defined in degree. Default is 0 meaning cue direction is from left to right along the x-axis.
	connection_graph	true,false	Whether to use a connection graph or not.
	segmentation_algorithm	string	Which segmentation algorithm to use. Default is CellposeSegmenter. For a list of available segmentation parameters see documentation.
	remove_small_objects_size	integer	Threshold for removing small objects from the instance segmentation mask.
	clear_border	true, false	If true, removes any segmentation that is not complete because the cell protrude beyond the edge of the image.
	keyfile_condition_cols	string	Only necessary for the run_key. Defines the column that holds the experiment condition information.
	save_sc_image	true,false	Whether to additionally plot the single cell images.
	extract_group_features	true,false	Whether to extract group features.
plot	plot_junctions	true, false	Indicates whether to create the junction polarity plot.
	plot_polarity	true, false	Indicates whether to create the polarity plot.
	plot_elongation	true, false	Indicates whether to create the elongation plot.
	plot_circularity	true, false	Indicates whether to create the circularity plot.
	plot_marker	true, false	Indicates whether to create the marker polarity plot.
	plot_ratio_method	true, false	Indicates whether to create the ratio plot.
	plot_shape_orientation	true, false	Indicates whether to create the shape orientation plot.
	plot_symmetry	true, false	Indicates whether to create the symmetry plot.
	plot_foi	true, false	Indicates whether to create the feature of interest plot.
	plot_sc_image	true, false	Indicates whether to create the single cell plots.
	plot_threshold_masks	true, false	Indicates whether to create a plot showing threshold masks.
	plot_sc_partitions	true, false	Indicates whether to plot individual partitioned cells in closeup.
	show_statistics	true, false	Add circular statistics to plot title.
	show_polarity_angles	true, false	Indicates whether to additionally add the polarity angles to the polarity plots.
	show_graphics_axis	true, false	Indicates whether to additionally add the axis to the plots.
	show_scalebar	true, false	Indicates whether to show a scalebar in visualizations or not.
	outline_width	integer	Outline width of a cell. Only affects visualization, not features. Default 2.
	length_scalebar_microns	float	Length of the scalebar in microns.
	graphics_output_format	png, pdf, svg, tif	The output format of the plot figures. Several can be specified. Default is png .
	dpi	integer	Resolution of the plots. Specifies the dots per inch. Default is 300.
	graphics_width	integer	The width of the output plot figures. Default 5.
	graphics_height	integer	The width of the output plot figures. Default 5.
	fontsize_text_annotations	integer	Size of text annotations in the plot.
	font_color	string	Font color. matplotlib font abbreviation.
	marker_size	true, false	Specify the marker size of all plots.
	alpha	true, false	Specify the alpha value of overlay masks.
	alpha_cell_outline	true, false	Specify cell outline alpha values.

Supplementary Table 10. All options and their input specification that can be specified in the parameters file sorted by parameter category.

Antibody name	Host species	Dilution	Product number/manufacturer
Anti-KLF4 antibody	*rabbit*	1:500	HPA002926 Sigma Aldrich
Human VE-Cadherin antibody	*goat*	1:1000	AF938 R&D Systems
Anti-GM130	*mouse*	1:500	610822 BD Biosciences
Phospho-SMAD 1/5/9	*rabbit*	1:500	13820S Cell Signaling
Notch1 (D1E11) XP®	*rabbit*	1:250	3608 Cell Signaling
Anti-Vimentin	*mouse*	1:100	V6389 Sigma Aldrich
Anti-Rabbit-Alexa-647	*donkey*	1:400	A31573 Invitrogen
Anti-Goat-Alexa-568	*donkey*	1:400	A11057 Invitrogen
Anti-Mouse-Alexa-488	*donkey*	1:400	A21202 Invitrogen
Anti-Rabbit-Alexa-488	*donkey*	1:400	A21206 Invitrogen

Supplementary Table 11. Table of primary and secondary antibodies.

Feature Name	Category	Feature	Description
neighbors_cell	toplogogy	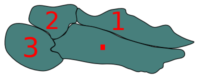	The absolute number of neighbors of the cell.
neighbors_mean_dif_1st	toplogogy	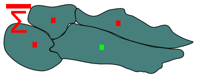	Mean difference of the feature of interest (FOI) to all first neighbors.
neighbors_median_dif_1st	toplogogy	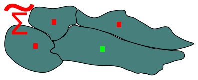	Median difference of the feature of interest (FOI) to all first neighbors.
neighbors_stddev_dif_1st	toplogogy	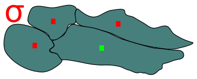	Standard derivation of the difference of the feature of interest (FOI) to all first neighbors.
neighbors_range_dif_1st	toplogogy	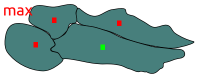	Maximal range of difference of the feature of interest (FOI) to all first neighbors.
neighbors_mean_dif_2nd	toplogogy	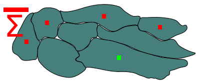	Mean difference of the feature of interest (FOI) to all second neighbors.
neighbors_median_dif_2nd	toplogogy	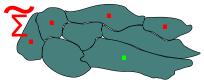	Median difference of the feature of interest (FOI) to all second neighbors.
neighbors_stddev_dif_2nd	toplogogy	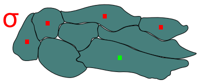	Standard derivation of the difference of the feature of interest (FOI) to all second neighbors.
neighbors_range_dif_2nd	toplogogy	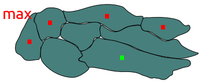	Maximal range of difference of the feature of interest (FOI) to all second neighbors.

Supplementary Table 12. Features targeting the cell neighborhood.

Feature Name	Category	Feature	Description
morans_i	toplogogy	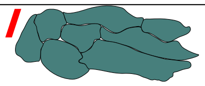	Statistical spacial correlation analysis
morans_p_norm	toplogogy	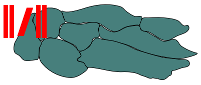	P-norm of the spacial correlation analysis.

Supplementary Table 13. Morans I group statistic performed on a feature of interest (FOI). This is an image wise statistic and is not reported cell wise.

Algorithm	Parameter	Options	Description
Cellpose	manually_annotated_mask	string	Naming suffix for (manually) annotated masks. Combine with use_given_mask.
	store_segmentation	true, false	Flag to allow storage of segmentation in the results folder.
	use_given_mask	true, false	Flag to load a segmentation mask or not. Segmentation mask should be in the same folder as the image to segment.
	model_type	string	Allowed Cellpose model types. ("cyto", "cyto2", "cyto3", "custom").
	model_type_nucleus	string	Allowed Cellpose model types for nucleus segmentation. ("nuclei", custom").
	model_path	string	Path to a custom model. Needs to be paired with model_type "custom".
	estimated_cell_diameter	int	Estimated cell diameter in pixels. Default 100.
	estimated_nucleus_diameter	int	Estimated nuclei diameter in pixels. Default 30.
	flow_threshold	float	Cellpose flow threshold value. Default 0.4.
	cellprob_threshold	float	Cellpose cell probability threshold value. Default 0.0.
	use_gpu	true, false	Indicates whether to use GPU or not.
	channel_cell_segmentation	string	Specify a concrete channel that is used for segmentation. Default is "channel_junction".
	channel_nuclei_segmentation	string	Specify a concrete channel that is used for nuclei segmentation. Default is "channel_nuclei".
DeepCell	segmentation_mode	string	Determines the segmentation mode. Either "whole-cell" or "nuclear".
	save_mask	true, false	Stores masks on disk in numpy format.
	maxima_threshold	float	To finetune specific and consistent errors in your data, this argument can be used during postprocessing. Lower values will result in more cells being detected. Higher values will result in fewer cells being detected.
	maxima_smooth	float	Controls what the model considers a unique cell. Lower values will result in more separate cells being predicted, whereas higher values will result in fewer cells.
	interior_threshold	float	Controls how conservative the model is in estimating what is a cell vs what is background. Lower values will result in larger cells, whereas higher values will result in smaller smalls.
	small_objects_threshold	integer	Minimal volume size in pixel before an object is detected as such.
	fill_holes_threshold	integer	Filling any holes that are contained in the predicted object up to a certain size.
	pixel_expansion	integer	Apply a manual pixel expansion after segmentation.
	channel_cell_segmentation	string	Specify a concrete channel that is used for segmentation. Default is "channel_junction".
	channel_nuclei_segmentation	string	Specify a concrete channel that is used for nuclei segmentation. Default is "channel_nuclei".
SAM	model_url	string	URL to a specific model.
	model_name	string	Name of the model. See https://github.com/facebookresearch/segment-anything for information.
	channel_cell_segmentation	string	Specify a concrete channel that is used for segmentation. Default is "channel_junction".
	channel_nuclei_segmentation	string	Specify a concrete channel that is used for nuclei segmentation. Default is "channel_nuclei".
	channel_organelle_segmentation	string	Specify a concrete channel that is used for organelle segmentation. Default is "channel_organelle".
microSAM	model_name	string	Model name to use. See https://computational-cell-analytics.github.io/micro-sam/micro_sam.html for information.
	checkpoint_path	string	Path to a checkpoint file that should be used.
	embedding_path	string	Path to the embedding file that should be used.
	pred_iou_thresh	float	Custom IO threshold.
	channel_cell_segmentation	string	Specify a concrete channel that is used for segmentation. Default is "channel_junction".
	channel_nuclei_segmentation	string	Specify a concrete channel that is used for nuclei segmentation. Default is "channel_nuclei".
	channel_organelle_segmentation	string	Specify a concrete channel that is used for organelle segmentation. Default is "channel_organelle".

Supplementary Table 14. Segmentation parameters for the supported segmentation algorithms. Most parameters are taken from the corresponding publication and/or github repository.

Deep learning	A family of machine-learning methods, based on deep neural networks (DNN), which are capable of learning representations from data with increasing levels of abstraction (4).
Deep Neural Network (DNN)	Computing system inspired by the biological neural networks consisting of multiple (deep) layers between the input and output layers.
Instance segmentation	Instance segmentation is a computer vision task that involves identifying and separating individual objects (e.g. biological cells or organelles) within an image, including detecting the boundaries of each object and assigning a unique labels. The result is a pixel-wise map of the image, where each pixel is associated with a specific object instance.
Polarity index	Length of the mean resultant vector computed by summing up all single orientation vectors and dividing by the number of measurements. The direction of the mean resultant vector mean angle of the circular distribution and its length the spread.
V-score	Similar to the polarity index, but also takes a pre-defined direction into account.
Application Programming Interface (API)	Generally, an API describes the connection between different computer programs or tools. It can also refer to everything an application programmer (e.g. python user of Polarity-JaM) needs to know about a piece of code and how to use it.
YAML, yaml or yml	A human-readable data serialisation language. Mainly used for configuration files and whenever data need to be stored or transmitted.

Supplementary Table 15. Glossary
